# Supplementary material for: Using a quantum work meter to test non-equilibrium fluctuation theorems
Source: Nat Commun. 2017 Nov 1;8:1241. doi: 10.1038/s41467-017-01308-7 (PMC5665923; doi:10.1038/s41467-017-01308-7)
Supplement: Supplementary file 1 — Supplementary Information [file 41467_2017_1308_MOESM1_ESM.pdf]

Supplementary Note 1 - Jarzynski identity and quantum work measurement

Work is the energy variation induced on a system $\mathcal{S}$ by a certain driving (the system is otherwise isolated). Suppose that the Hamiltonian of $\mathcal{S}$ changes from an initially H to a final $\tilde{H}$ and that the system is driven by a certain unitary operator $\mathcal{U}_{\mathcal{S}}$ in between. We denote the eigenvalues and eigenvectors of H as E_n and $|\varphi_{n,\alpha}\rangle$ (where α labels different eigenstates with the same energy). In turn the eigenvalues and eigenvectors of $\tilde{H}$ are denoted as $\tilde{E}_m$ and $|\tilde{\varphi}_{m,\gamma}\rangle$ (again, γ labels states with the same energy $\tilde{E}_m$). Suppose that we measure the energy at the initial and final times. The work probability distribution $P(w)$ is nothing but the probability density to obtain a value w as the difference between the results of the two energy measurements. This can obviously be computed as

$$P(w) = \sum_{n,m} p_n p_{m|n}\, \delta\left[w - (\tilde{E}_m - E_n)\right], \tag{1}$$

where p_n is the probability of initially measuring energy E_n and $p_{m|n}$ is the probability of measuring energy $\tilde{E}_m$ at the end of the driving given that E_n was detected at the beginning. If the initial state of $\mathcal{S}$ is ρ, the above probabilities can be simply written in terms of the projectors $\Pi_n = \sum_\alpha |\varphi_{n,\alpha}\rangle\langle\varphi_{n,\alpha}|$ and $\tilde{\Pi}_n = \sum_\gamma |\tilde{\varphi}_{n,\gamma}\rangle\langle\tilde{\varphi}_{n,\gamma}|$. Thus,

$$p_n = \mathrm{Tr}(\rho\,\Pi_n), \qquad p_{m|n} = \frac{1}{p_n}\,\mathrm{Tr}(\tilde{\Pi}_m \mathcal{U}_{\mathcal{S}} \Pi_n \rho\, \Pi_n \mathcal{U}_{\mathcal{S}}^\dagger). \tag{2}$$

Jarzynski identity follows immediately from the above definition of the work probability distribution. In fact, if we compute the exponential average of the work, we get

$$\begin{aligned} \langle e^{-\beta w}\rangle &= \int dw P(w) e^{-\beta w} \\ &= \sum_{n,m} p_n p_{m|n} e^{-\beta(\tilde{E}_m - E_n)}. \end{aligned} \tag{3}$$

If the initial state is thermal, then $\rho = \sum_n \Pi_n e^{-\beta E_n}/Z$ (where Z is the partition function $Z = \sum_n g_n e^{-\beta E_n}$ with $g_n = \mathrm{Tr}(\Pi_n)$ being the degeneracy of level E_n). Then, we can replace $p_n = e^{-\beta E_n}/Z$ and perform the summation over the label n by noticing that $\sum_n p_{m|n} = \mathrm{Tr}(\tilde{\Pi}_m) = \tilde{g}_m$. In this way we obtain

$$\langle e^{-\beta w}\rangle = \frac{1}{Z}\sum_m \mathrm{Tr}(\tilde{\Pi}_m) e^{-\beta\tilde{E}_m} = \frac{\tilde{Z}}{Z},$$

where the partition function of the final Hamiltonian is $\tilde{Z} = \sum_m \tilde{g}_m e^{-\beta\tilde{E}_m}$, with $\tilde{g}_m = \mathrm{Tr}(\tilde{\Pi}_m)$ the degeneracy of level $\tilde{E}_m$. The final step that leads to the Jarzynski identity is simply to notice that for a Gibbs state we have $e^{-\beta\Delta F} = \tilde{Z}/Z$. Thus, the average exponential work becomes independent of the intermediate driving process $\mathcal{U}_{\mathcal{S}}$ and turns out to be determined by equilibrium properties. Thus, $\langle e^{-\beta w}\rangle = e^{-\beta\Delta F}$.

When $\mathcal{S}$ has a finite dimensional Hilbert space, w can only take discrete values. In this case, instead of using the probability density we use directly the probability for each value of w. Thus, we can arrange $P(w)$ as a vector with D^2 components. Taking into account the normalisation condition, the vector of independent probabilities is $D^2 - 1$ dimensional. In turn, being the Jarzynski identity a linear equation in terms of $P(w)$ it constraints the probability vector to belong to a $D^2 - 2$ dimensional hyperplane. But, of course, there are further constrains reducing the number of independent probabilities. The simplest way to obtain this number is to go back to the definition of $P(w)$ in Supplementary Equation (1) and count the number of free parameters we have. In this equation, the probabilities p_n are fixed by the initial state. Then, the number of independent probabilities is determined by the number of independent parameters in $p_{m|n}$. For the non-degenerate case we consider here (where all the values of work are different), the calculation is simple. In fact, the coefficients $p_{m|n}$ form a doubly stochastic matrix (since they are all positive numbers such that $\sum_n p_{m|n} = \sum_m p_{m|n} = 1$). For such square matrix of dimension D, there is always $(D-1)^2$ free parameters. Indeed, this is the dimensionality of the manifold of where the probability vector lies. Jarzynski identity establishes that this manifold is a β-dependent hyperplane (a line in our case, where $D = 2$).

Supplementary Note 2 - Work measurement as a POVM

Let us consider a system $\mathcal{S}$ with a D-dimensional space of states and show, in a simple way, that the work measurement can be viewed as a generalised quantum measurement. For this, we start by writing the probability for

a given value of work $w_{nm} = \tilde{E}_m - E_n$ as

$$P(w_{nm}) = p_{m|n}\, p_n. \tag{4}$$

Using the formula for the transition probability, we find that

$$P(w_{nm}) = \mathrm{Tr}(\tilde{\Pi}_m \mathcal{U}_{\mathcal{S}} \Pi_n \rho \Pi_n\, \mathcal{U}_{\mathcal{S}}^{\dagger}) = \mathrm{Tr}(\rho A_{nm}), \tag{5}$$

where

$$A_{nm} = \Pi_n \mathcal{U}_{\mathcal{S}}^{\dagger} \tilde{\Pi}_m \mathcal{U}_{\mathcal{S}} \Pi_n. \tag{6}$$

It is simple to verify that the operators A_{nm} expand the identity as $\mathbb{I} = \sum_{n,m} A_{nm}$ and that they are positive semi-definite (i.e., that for any state $|\chi\rangle$ we have $\langle\chi|A_{nm}|\chi\rangle \geq 0$). Therefore, the operators A_{nm} define a positive operator valued measure (POVM), which is the most general type of measurement one can perform in quantum mechanics. Therefore, work can be measured in the same way as any POVM can: A powerful result (Neumark's theorem) establishes that any POVM can be realised by coupling the system $\mathcal{S}$ with an ancillary system $\mathcal{A}$ and then performing a standard projective measurement on $\mathcal{A}$. This measurement can be performed at a single time. Thus, surprisingly, the two-time work measurement strategy can be replaced by a single-time strategy (which is the basic idea exploited by our QWM). In the following section we show how one can construct an approximation for that ideal apparatus, that we call Quantum Work Meter (QWM).

Supplementary Note 3 - Probability distribution for the outcome of a QWM

Here we compute the probability distribution for the result of the measurement of the auxiliary register of a general Quantum Work Meter. The protocol defining the apparatus is shown in Figure 1 of the main text. A system $\mathcal{S}$ is coupled to an ancillary one $\mathcal{A}$. This ancilla is a continuous variable system (of course this can be relaxed). The system $\mathcal{S}$ and the ancilla $\mathcal{A}$ are subject to the following evolution: *i*) an entangling interaction U is applied (which correlates $\mathcal{S}$ and $\mathcal{A}$), *ii*) the evolution $\mathcal{U}_{\mathcal{S}}$ is applied on the system $\mathcal{S}$, *iii*) a second entangling interaction $\tilde{U}$ is applied. Finally, after this sequence $\mathcal{A}$ is measured. The initial state of the system formed by $\mathcal{S}$ will be assumed to be a product state. For simplicity, we first assume that the states are pure and denote them as $|\xi\rangle$ (the state of $\mathcal{S}$) and $|\phi\rangle$ (the state of $\mathcal{A}$). We will later generalise the result for an initial state which is a tensor product of arbitrarily mixed states. After the sequence of operations we described above, the total final state is:

$$|\Phi(t_f)\rangle = \tilde{U}\,(I_{\mathcal{A}} \otimes \mathcal{U}_{\mathcal{S}})\, U\, |\phi\rangle \otimes |\xi\rangle$$

The nature of the two entangling operations, that was discussed in the main text, is such that they both induce translations of $\mathcal{A}$ which depend on the state of $\mathcal{S}$. More specifically, $U = e^{-\frac{i}{\hbar}\lambda \hat{z}_{\mathcal{A}} \otimes H}$ and $\tilde{U} = e^{\frac{i}{\hbar}\lambda \hat{z}_{\mathcal{A}} \otimes \tilde{H}}$, where we use $\hat{z}_{\mathcal{A}}$ to denote the generator of translations of $\mathcal{A}$ along a certain variable p. Using this, it is simple to rewrite the final state as

$$|\Phi(t_f)\rangle = \sum_{n,m} D_{nm}|\phi\rangle \otimes \tilde{\Pi}_m \mathcal{U}_{\mathcal{S}} \Pi_n |\xi\rangle$$

In this equation the displacement operators D_{nm} act on the states of $\mathcal{A}$ and are defined as $D_{nm} = e^{\frac{i}{\hbar}\lambda w_{nm} \hat{z}_{\mathcal{A}}}$.

The interpretation of the above equation is simple: After the sequence of operations, $\mathcal{S}$ and $\mathcal{A}$ become entangled in such a way that a record of w_{nm} is stored in $\mathcal{A}$. The states $|F_{nm}\rangle \equiv D_{nm}|\phi\rangle$ are "flag states" associated with the different values of work. When these states are orthogonal, they can be unambiguously distinguished and the value of work can be retrieved. Below, we will consider a more realistic scenario where the initial state of $\mathcal{A}$ is a localised coherent state. In that case, the flag states are displaced coherent states (which are simply translated along the variable p direction by an amount that is proportional to w_{nm}). These states are not strictly orthogonal, but have a finite overlap. This induces an error in the work estimation protocol. However, the error can be exponentially reduced by simply increasing the interaction strength λ (as the overlap exponentially decreases with λ).

From the above expression it is simple to obtain the quantum state of $\mathcal{A}$ by computing its reduced density matrix (which is obtained from the total state by tracing out the system $\mathcal{S}$). Thus,

$$\rho_{\mathcal{A}}(t_f) = \sum_{n,n',m} \mathrm{Tr}\left(\tilde{\Pi}_m \mathcal{U}_{\mathcal{S}} \Pi_n |\xi\rangle\langle\xi| \Pi_{n'} \mathcal{U}_{\mathcal{S}}^{\dagger}\right)\, D_{nm}|\phi\rangle\langle\phi| D_{n'm}^{\dagger}. \tag{7}$$

This expression can be generalised to the case where the initial states of $\mathcal{S}$ and $\mathcal{A}$ are initially mixed. In fact, if $\rho_{\mathcal{S}}$ and $\rho_{\mathcal{A}}$ respectively denote the initial density matrices of $\mathcal{S}$ and $\mathcal{A}$, the final state of $\mathcal{A}$ is

$$\rho_{\mathcal{A}}(t_f) = \sum_{n,n',m} \mathrm{Tr}\left(\tilde{\Pi}_m \mathcal{U}_{\mathcal{S}} \Pi_n \rho_{\mathcal{S}} \Pi_{n'} \mathcal{U}_{\mathcal{S}}^\dagger\right) D_{nm} \rho_{\mathcal{A}} D_{n'm}^\dagger. \tag{8}$$

From the above equation we obtain the probability density for detecting the value p in a measurement of $\mathcal{A}$. Thus,

$$P_{\mathcal{A}}(p) = \sum_{n,n',m} \mathrm{Tr}\left(\tilde{\Pi}_m \mathcal{U}_{\mathcal{S}} \Pi_n \rho_{\mathcal{S}} \Pi_{n'} \mathcal{U}_{\mathcal{S}}^\dagger\right) \langle p - \lambda w_{nm} | \rho_{\mathcal{A}} | p - \lambda w_{n'm} \rangle. \tag{9}$$

The contribution of the diagonal ($n = n'$) and off-diagonal ($n \neq n'$) terms play a different role in the above expression. In fact, it is simple to show that the diagonal contribution is a smeared version of the true work distribution. Thus,

$$\sum_{n,m} \mathrm{Tr}\left(\tilde{\Pi}_m \mathcal{U}_{\mathcal{S}} \Pi_n \rho_{\mathcal{S}} \Pi_n \mathcal{U}_{\mathcal{S}}^\dagger\right) \langle p - \lambda w_{nm} | \rho_{\mathcal{A}} | p - \lambda w_{nm} \rangle = \int dw\, P(w)\, f(p - \lambda w), \tag{10}$$

where the window function is

$$f(p) = \langle p | \rho_{\mathcal{A}} | p \rangle. \tag{11}$$

Therefore, the off-diagonal terms of $\rho_{\mathcal{S}}$ are responsible for the error in the work estimation and should be made small for it to be accurate. It is simple to show that if the momentum wave function of the initial state $|\phi\rangle$ is a Gaussian with a momentum dispersion $1/\sigma$ (σ is the position dispersion), then the off-diagonal terms are bounded by:

$$\left| \sum_{n\neq n',m} \mathrm{Tr}\left(\tilde{\Pi}_m \mathcal{U}_{\mathcal{S}} \Pi_n \rho_{\mathcal{S}} \Pi_{n'} \mathcal{U}_{\mathcal{S}}^\dagger\right) \langle p - \lambda w_{nm} | \phi \rangle \langle \phi | p - \lambda w_{n'm} \rangle \right| \leq \sum_{n\neq n',m} \left| \mathrm{Tr}\left(\tilde{\Pi}_m \mathcal{U}_{\mathcal{S}} \Pi_n \rho_{\mathcal{S}} \Pi_{n'} \mathcal{U}_{\mathcal{S}}^\dagger\right) \right| \frac{\sigma}{\hbar\sqrt{\pi}} e^{-\frac{\sigma^2\lambda^2}{4\hbar^2}(E_n - E_{n'})^2}$$

Thus, by increasing λ (the interaction strength) or σ (the position dispersion of the initial state) we exponentially reduce the error in the work estimation. It is worth noting that by increasing σ we reduce the momentum uncertainty and localise the initial state in momentum. Naturally, the method becomes precise when the initial localisation in momentum is much smaller than the difference between the first momentum kicks (which is fixed by the product $\lambda(E_n - E_{n'})$). When these conditions are satisfied, the off-diagonal terms can be neglected and the probability density to detect p is

$$P_{\mathcal{A}}(p) = \int dw\, P(w)\, f(p - \lambda w),$$

where the window function $f(p)$ is defined as $f(p) = |\langle p|\phi\rangle|^2$ (which, for a coherent state is simply $f(p) = e^{-\frac{\sigma^2}{\hbar^2}p^2} \sigma/\hbar\sqrt{\pi}$).

QWM using an atom chip

Here we consider the implementation of the QWM using a cloud of atoms in a chip. As described in the main text, $\mathcal{S}$ is the pseudo spin 1/2 associated with the $F = 2$, $m_F = 1, 2$ hyperfine states of a ^{87}Rb atom (which, as discussed in the main text, behaves as a two level atom). $\mathcal{A}$ is encoded in the motional degrees of freedom of each atom. There are two subtle differences between the ideal protocol for a QWM described in the previous section and the implementation in an atom chip. The first different concerns the final measurement. Thus, in the previous section we computed the probability for a final momentum measurement but in the real experiment we are forced to measure the atomic position by taking an image of the atomic clouds. Therefore we will show below that the position measurement enables us to determine work and sample $P(w)$. The second difference is that in the real experiment we should take into account the fact that the ancilla $\mathcal{A}$ evolves during the whole process because the atoms actually move (they freely fall along the vertical direction). Thus, the real protocol describing the experiment is shown in Fig. 1.

Let us now analyse this process. We can first neglect the free fall taking place between the two entangling operations (we take this into account later) and assume the initial state of $\mathcal{A}$ is a coherent state localised around initial values of position and momentum which we arbitrarily take as $z = 0$ and $p = 0$. We denote this state as $|\phi\rangle = |0, 0\rangle$.

The calculation presented in the above section should be slightly modified. In this case the flag states are $|F_{nm}\rangle = u^{(1)}_{fall} D_{nm}|0,0\rangle$. Taking into account that $u_{fall} = e^{-\frac{i}{\hbar}t(\hat{p}^2_{\mathcal{A}}/2m_a - m_a g \hat{z}_{\mathcal{A}})}$ (where t is the duration of the free-fall, m_a is the mass of the atoms, and g the gravity acceleration) we can easily compute the expectation value of the position for each flag state (as well as the corresponding position dispersion). In fact, we have

$$z_{nm}(t) = -w_{nm}\,\frac{\lambda t}{m_a} + \frac{g}{2}t^2 \qquad \text{and} \qquad \Delta z_t = \frac{\sigma}{\sqrt{2}}\sqrt{1+\left(\frac{\hbar\, t}{m_a \sigma^2}\right)^2}. \tag{12}$$

Therefore, we can notice that by measuring the final position of the atoms we can infer the value of the momentum before the fall and thus acquire information about work w. In fact, the difference between the positions of the clouds is proportional to the difference in the values of work. The price we have to pay, is that the spread of the wave packets increases during the free-fall.

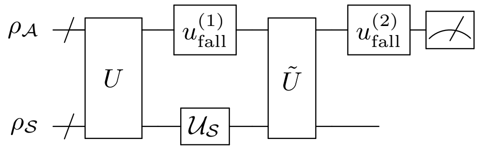

Supplementary Figure 1: Set of gates describing the atom chip QWM. $u^{(1)}_{\rm fall}$ and $u^{(2)}_{\rm fall}$ are the free fall evolution that the atoms feel during the experiment. Finally there is a measurement of the position of the atoms.

In a more realistic description of the experiment, we need to include also the free fall between the entangling gates ($u^{(1)}_{\rm fall}$ in the Supplementary Figure 1). It is easy to verify that since $u^{(1)}_{\rm fall}\,\hat{z}_{\mathcal{A}}\,u^{(1)\dagger}_{\rm fall} = \hat{z}_{\mathcal{A}} + \hat{p}_{\mathcal{A}}\,t/m_a + \mathbb{I}_{\mathcal{A}}\,g\,t^2/2$ (where t is the duration of the free fall), then $u^{(1)}_{\rm fall}\,e^{\frac{i}{\hbar}\lambda\,\hat{z}_{\mathcal{A}}\otimes\tilde{H}}\,u^{(1)\dagger}_{\rm fall} = e^{\frac{i}{\hbar}\lambda\,\hat{z}_{\mathcal{A}}\otimes\tilde{H}}e^{\frac{i}{\hbar}\frac{t}{m}\lambda\,\hat{p}_{\mathcal{A}}\otimes\tilde{H}}e^{\frac{i}{\hbar}\theta\,\mathbb{I}_{\mathcal{A}}\otimes\tilde{H}}$. Thus, the first term is the usual entangling operation and the last term is just a phase depending on the value of the energy. In turn, the second term is an entangling operation where the atom is displaced along position (instead of momentum) depending on the state of $\mathcal{S}$. In summary, the free-fall in between the entangling gates simply induces an extra translation of the atoms by an amount that depends on the final value of the energy.

Finally, we show in the Supplementary Figure 2 the distribution probability that we obtain in each experiment. In the plots we show the projections of the Jarzynski manifold, that appears in Fig. 2 (a) of the main text, onto the different axes.

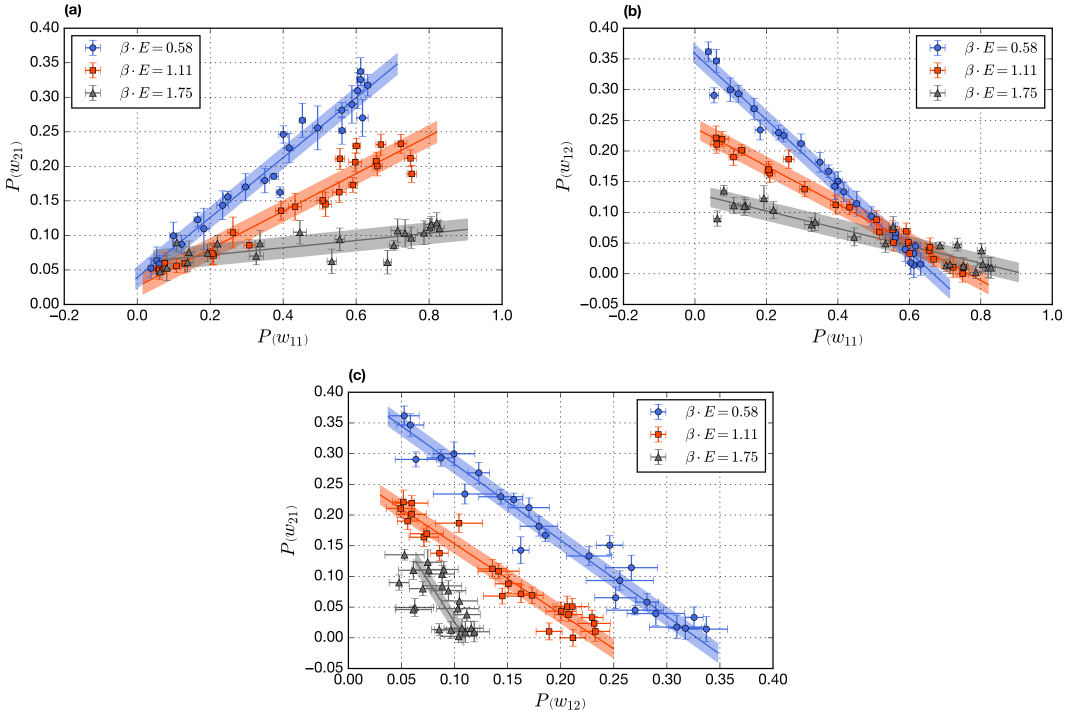

Supplementary Figure 2: Measured work probabilities. **(a)–(c)** Each point defines a probability vector (with its experimental error) measured for a certain driving. Error bars are the SEM of three independent experiments. The plots are the projections of the Jarzynski manifold, that appears in Fig. 2 (a) of the main text, onto the different axes. The three lines correspond to three temperatures: $\beta E = 0.58 \pm 0.02$ (blue circle), 1.11 ± 0.02 (red square), 1.75 ± 0.04 (grey triangle). For each temperature all points lie in the same Jarzynski manifold (which in this case is a line).
